# The Impact of Glucagon-like Peptide-1 Receptor Agonists on Cardiovascular–Kidney–Metabolic Health in Romanian Patients with Type 2 Diabetes: A Retrospective Study

**DOI:** 10.3390/jcm15010152

**Published:** 2025-12-25

**Authors:** Niculina Lixandru, Laura Gaita, Simona Popescu, Andreea Herascu, Bogdan Timar, Romulus Timar

**Affiliations:** 1 Doctoral School of Medicine, “Victor Babes” University of Medicine and Pharmacy, 300041 Timisoara, Romania; 2 Second Department of Internal Medicine, “Victor Babes” University of Medicine and Pharmacy, 300041 Timisoara, Romania; 3 Department of Diabetes, “Pius Brînzeu” Emergency County Hospital, 300723 Timisoara, Romania; 4 Centre for Molecular Research in Nephrology and Vascular Disease, “Victor Babes” University of Medicine and Pharmacy, 300041 Timisoara, Romania

**Keywords:** type 2 diabetes, glucagon-like peptide-1 receptor agonists, cardiovascular risk factors, weight loss, insulin resistance, kidney function, cardiovascular–kidney–metabolic health

## Abstract

**Background/objectives:** Chronic degenerative complications of diabetes, such as atherosclerotic cardiovascular disease and chronic kidney disease, contribute to an increased morbimortality in patients with type 2 diabetes (T2D), and thus, a multifactorial approach becomes essential. Among the classes of antihyperglycemic agents with beneficial pleiotropic cardiorenal effects, the glucagon-like peptide-1 receptor agonists (GLP-1 RAs) have proven to reduce the risk of major adverse cardiovascular (CV) and renal events. This study aims to assess the impact of treatment with GLP-1 RA on CV risk factors, insulin sensitivity, and renal function in Romanian patients with T2D. **Methods:** In an observational retrospective study, 150 patients with T2D were evaluated at the start of therapy with a GLP-1 RA and then after 6 and 12 months. **Results:** After 12 months of treatment, 59.3% of patients succeeded in achieving weight loss of over 5% of their initial weight, and 24.7% of patients achieved weight loss of over 10% of their initial weight, with the most significant decrease being measured in the first 6 months. HbA1c has shown a similar profile, with a significant reduction in the first 6 months of treatment, continued at a slower rate in the following 6 months. Additionally, the lipid profile, blood pressure values, and uric acid values, alongside the triglyceride/high-density lipoprotein cholesterol (TG/HDLc) ratio and the triglyceride–glucose (TyG) index have improved in these T2D patients treated with GLP-1 RA, while their eGFR decrease was slower than the one expected for similar populations without such a pharmacologic agent in their regimen. **Conclusions:** Treatment with GLP-1 RA in patients with T2D is associated with an improved cardiovascular–kidney–metabolic risk profile, ameliorated glycemic control, reduced weight, lower insulin resistance, and slower kidney disease progression.

## 1. Introduction

Diabetes is a chronic metabolic condition with a significant global socio-economic impact through the more than 588 million patients worldwide in 2024, numbers that are expected to increase to more than 852 million patients in 2050 [1]. A significant part of its burden is represented by the chronic degenerative complications that contribute to an increased morbimortality. Atherosclerotic cardiovascular disease (ASCVD) has a higher prevalence in patients with type 2 diabetes (T2D), a condition which is usually associated with multiple risk factors such as overweight or obesity, insulin resistance, hypertension, or dyslipidemia [2]. Additionally, chronic kidney disease (CKD) is present in approximately 20–40% of patients with T2D, occasionally even from the apparent diagnosis of this condition, and it impairs the prognosis of these patients either through the development of end-stage kidney disease or through an increased risk of CV events [3].

In this context, it becomes evident that a multifactorial approach is essential for patients with T2D in order to prevent the development and/or progression of ASCVD and CKD. One of the antihyperglycemic pharmacologic agents that has proven pleiotropic beneficial effects are glucagon-like peptide-1 receptor agonists (GLP-1 RAs) that, alongside their efficacy in improving glycemic control, without an additional risk of hypoglycemia, by increasing the secretion of insulin and reducing the secretion of glucagon in a manner dependent on blood glucose levels, contribute to weight loss through reducing appetite, increasing satiety, and delaying gastric emptying. Additionally, it has been shown that GLP-1 RAs exert favorable cardiorenal effects, improving insulin sensitivity, preventing endothelial dysfunction, lowering blood pressure values, improving coronary flow, improving myocardial contractility, reducing systemic inflammation, decreasing oxidative dress, ameliorating the lipid profile, increasing natriuresis, reducing hyperfiltration, and improving tubuloglomerular feedback [4,5,6].

The CV safety and benefits of GLP-1 RAs have been shown in multiple cardiovascular outcome trials (CVOTs) such as LEADER (liraglutide), SUSTAIN-6 (once-weekly injectable semaglutide), PIONEER-6 (oral semaglutide), and REWIND (dulaglutide) and most recently SOUL (oral semaglutide), with results that have positioned this class of antihyperglycemic drugs as first-line choices, in the absence of contraindications, in the treatment of patients with T2D and ASCVD or indicators of high CVD risk, independently of glucose control. Moreover, their very high or high efficacy in weight loss and achievement and maintenance of glycemic goals have led to indicating GLP-1 RA as a key therapy in the management of patients with T2D [7,8,9,10,11].

Another unexpected effect of this class of antihyperglycemic agents is related to renal protection, as their use has been associated with a reduction in albuminuria and a slower decrease in kidney function, as shown in the FLOW trial (once-weekly injectable semaglutide), the first dedicated kidney trial with a GLP-1 RA [12,13]. These cardiorenal protective effects have been summarized in a recent systematic review and meta-analysis which showed that long-acting GLP-1 RAs reduce the risk of major adverse CV events, all-cause mortality, composite-kidney outcome, and even hospitalization for heart failure, without any significant heterogeneity driven by the subcutaneous or oral route of administration [14].

In this context, with an abundance of positive results from different types of studies, yet with a reduced representation of countries from Central–Eastern Europe such as Romania and with reduced data from real-world trials, we aimed to evaluate the impact on CV risk factors, insulin resistance, and renal function of the treatment with GLP-1 RAs in patients with T2D.

## 2. Materials and Methods

### 2.1. Study Design and Patients

This observational, retrospective study enrolled, consecutively, 150 patients (79 women and 71 men) diagnosed with T2D and treated with GLP-1 RA who were hospitalized in the Department of Diabetes, Nutrition and Metabolic Diseases of the “Pius Brînzeu” Emergency County Hospital in Timișoara, Romania and then monitored for a period of 1 year, with the assessment of biological parameters at the initiation of treatment, at 6 months, and at 12 months. All of the 150 patients completed the 12 months of follow-up without any discontinuations of GLP-1 RA treatment and without missing data. The protocol, designm and informed consent of this study were approved by the Ethics Committee of the hospital (no. 422/28.02.2024). All patients provided written informed consent, while the research was carried out according to the principles of the Declaration of Helsinki. A previous diagnosis of T2D and an age > 18 years represented the inclusion criteria, while the exclusion criteria were an estimated glomerular filtration rate (eGFR) < 30 mL/min/1.73 m^2^ or the inability to provide an adequate medical history or informed consent. The patients were treated with either semaglutide administered subcutaneously, once weekly (0.5 or 1 mg); semaglutide administered orally, once daily (7 or 14 mg); or dulaglutide administered subcutaneously, 1.5 mg, once weekly.

### 2.2. Clinical, Anthropometric, and Laboratory Data

The patients’ gender, age, age at the diagnosis of T2D, height, weight, associated comorbidities, and the presence of diabetes complications were collected from the medical charts. Weight was measured using standardized equipment and procedures. The body mass index (BMI) was assessed as weight (kg)/height^2^ (m^2^). Hypertension was defined as systolic blood pressure > 140 mmHg, diastolic blood pressure > 90 mmHg, or treatment with antihypertensive agents, while the measurements were performed according to the European Society of Cardiology guideline recommendations [15]. eGFR was calculated using the CKD-EPI creatinine equation, while the presence and stage of CKD were established depending on the eGFR and urinary albumin/creatinine ratio (UACR) [16]. Additionally, fasting blood glucose (FBG), hemoglobin A1c (HbA1c), total cholesterol (TC), low-density lipoprotein cholesterol (LDLc), triglycerides (TG), high-density lipoprotein cholesterol (HDLc), uric acid (UA), creatinine, urinary albumin, and urinary creatinine were assessed using standardized methods in the laboratory of the “Pius Brînzeu” Emergency County Hospital in Timișoara, after at least 12 h of fasting, and postprandial blood glucose was determined using a glucose meter. In order to assess insulin resistance, validated surrogate markers were used, namely the triglyceride/high-density lipoprotein cholesterol (TG/HDLc) ratio and the triglyceride–glucose index (TyG) calculated as ln [fasting triglycerides (mg/dL) × fasting plasma glucose (mg/dL)/2] [17].

### 2.3. Statistical Analysis

MedCalc^®^ Statistical Software version 20.210 was used for the statistical analysis of the collected data (MedCalc Software Ltd., Ostend, Belgium; https://www.medcalc.org (accessed on 17 January 2025); 2022). The continuous variables with Gaussian distribution are presented as mean ± standard deviation; continuous variables with non-parametric distribution as median, [interquartile range], and (average rank); and categorical variables as frequencies and percentages. The significance of differences between two groups was assessed with the Mann–Whitney U test in non-parametric populations, Student’s *t*-test in Gaussian populations, and with the chi-squared test in categorical populations, while the Friedman test was used to determine the significance of differences between 3 groups. Additionally, the significance of the median difference between two paired samples was assessed with the paired-samples Wilcoxon test. The sample size was calculated before the inclusion of subjects, considering data found in the literature, in order to obtain a confidence level of 95% and a statistical power higher than 80%. The *p*-value threshold below which the statistical significance was considered is 0.05.

## 3. Results

After taking into consideration the inclusion and exclusion criteria, the study group was represented by 150 patients with T2D, of whom 79 (52.7%) were women and 71 (47.3%) were men. A detailed overview of the studied sample’s characteristics, compared by sex, is presented in Table 1.

A statistically significant difference was shown regarding the initial weight (102.00 kg vs. 97.00 kg in men vs. women, *p* = 0.016), HDLc (36.00 mg/dL vs. 41.00 mg/dL in men vs. women, *p* < 0.0001), and uric acid (5.52 mg/dL vs. 4.92 mg/dL in men vs. women, *p* = 0.002). Additionally, it was shown that men had a statistically significant higher prevalence of peripheral artery disease vs. women (33.8% vs. 13.9%, *p* = 0.004). This comparison between genders regarding chronic degenerative complications of diabetes and comorbidities can be found in Table 2.

**Figure 1:**
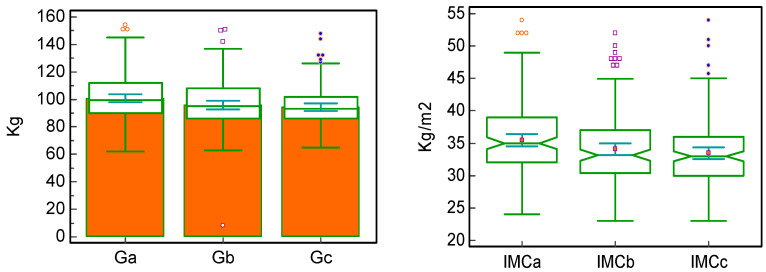
Evolution of weight and BMI (body mass index)—median, [interquartile range], and (mean rank)—during treatment with GLP-1 RA. a = initially, b = after 6 months, c = after 12 months. G = weight, IMC = BMI. GLP-1 RA—glucagon-like peptide-1 receptor agonist.

**Figure 2:**
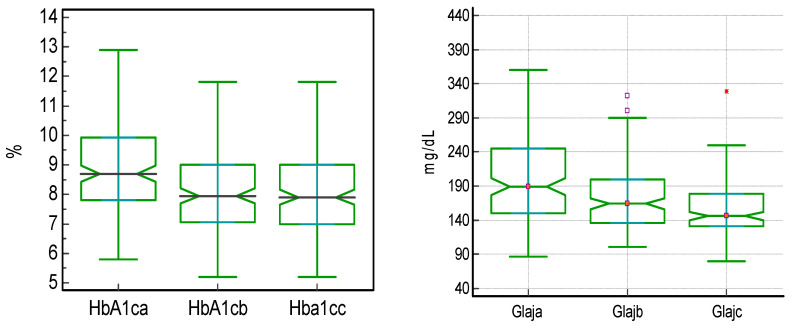
Evolution of HbA1c (hemoglobin A1c) and fasting plasma glucose (FPG)—median, [interquartile range], and (mean rank)—during treatment with GLP-1 RA. a = initially, b = after 6 months, c = after 12 months. Glaj = FPG. GLP-1 RA—glucagon-like peptide-1 receptor agonist.

Out of a total of 150 patients, 72 (48.0%) received treatment with semaglutide administered subcutaneously, 43 (28.6%) with semaglutide administered orally, and 35 (23.3%) with dulaglutide. This treatment has been associated with statistically significant decreases in weight, BMI, HbA1c, and FPG between the initial moment and after 6 months of treatment and between 6 and 12 months of treatment and also between the initial moment and after 12 months of treatment, with the most significant differences being noticed in the initial 6 months. These differences are shown in Table 3 and Figure 1 and Figure 2.

Out of a total of 150 patients, 89 (59.3%) succeeded in achieving weight loss of over 5% of their initial weight, and 37 patients (24.7%) achieved weight loss of over 10% of their initial weight, after 12 months of treatment with a GLP-1 RA. Additionally, from an initial number of 15 (10.0%) patients with a HbA1c < 7%, 41 (27.3%) attained the treatment target after 12 months of treatment with a GLP-1 RA, a statistically significant difference (*p* = 0.017, calculated using the chi-squared test).

Moreover, the 12 months of treatment with GLP-1 RA contributed to statistically significant beneficial changes regarding LDLc, HDLc, TG, SBP, DBP, UA, and eGFR, results that are shown in Table 4, with variable clinical relevance according to the parameter and dimension of the change in these levels.

**Figure 3:**
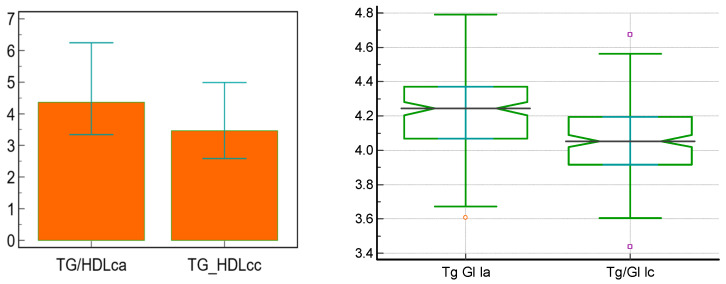
Evolution of insulin resistance markers—median, [interquartile range]—during treatment with GLP-1 RA. a = initially, c = after 12 months. GLP-1 RA—glucagon-like peptide-1 receptor agonists, Tg/HDLc—triglyceride/high-density lipoprotein cholesterol ratio, Tg Gl—triglyceride–glucose index.

Lastly, 12 months of treatment with GLP-1 RA contributed to statistically significant decreases in insulin resistance biomarkers, as shown in Table 5 and Figure 3.

## 4. Discussion

### 4.1. Findings and Their Interpretation

This research revealed statistically significant differences between male and female participants in terms of initial weight, serum uric acid, and prevalence of peripheral artery disease (higher in men) and HDLc (higher in women), results similar to those of previous studies [18,19].

Regarding the potential impact of treatment with GLP-1 RAs, 59.3% of patients had a weight reduction of more than 5% during the entire study duration, and 24.7% had a weight decrease of 10% after 12 months of treatment, effects similar to those described in the PIONEER 5 clinical trial, although it had a radically different design [20]. The absolute weight loss differences between month 6 and the initial dose (an average of 4 kg), month 12 vs. month 6 (an average of 2 kg), and between month 12 and the initial dose (an average of 6 kg) indicate a sustainable reduction in body weight, with significant early beneficial results and with the main weight loss being measured in the first months of the treatment. These results are similar to those observed in the SUSTAIN-1 clinical trial, in which the weight loss was of approximately 3.73 kg with injectable semaglutide (0.5 mg/week) and 4.53 kg with injectable semaglutide 1 mg/week, and those observed in the AWARD-3 clinical trial, which highlighted a decrease of 3.1 kg in 12 months of treatment with dulaglutide (1.5 mg/week) [21,22].

Additionally, the absolute decreases in BMI between month 6 and the initial dose (an average of 1.1 kg/m^2^), month 12 vs. month 6 (an average of 0.8 kg/m^2^), and between month 12 and the initial dose (an average of 2 kg/m^2^) are consistent with those of the weight loss per se, suggesting a significant effect in reducing the metabolic risk of patients treated with GLP-1 RAs, effects already noticed in the first half of the trial duration. These findings can also explain the beneficial effects that treatment with GLP-1 RA had on insulin resistance, with a statistically significant reduction in TG/HDLc ratio (with an average of 1.04) and in TyG index (with an average of 0.16), both surrogate markers of insulin resistance, suggesting improved insulin sensitivity as also shown in multiple other trials and meta-analyses [23,24,25].

From the point of view of HbA1c, the results are similar, with the most significant reduction being noticed in the first 6 months of treatment, 0.7%, with a total average decrease of 0.8% in the 12 months of study, indicating a sustainable and significant glycemic effect without increasing the risk of hypoglycemia, similarly to what was identified in the PIONNER 5 clinical trial [20]. Moreover, though at the beginning of the treatment with GLP-1 RA, only 10% of the patients had reached the HbA1c target of <7%, after 12 months of treatment, 27.3% reached the target without any additional interventions, results underlying the favorable impact of using this class of antidiabetic drugs. These effects have been also noticed regarding the values of FBG, which decreased statistically significantly in the first 6 months, with approximately 15 mg/dL; in the following 6 months, with 11.5 mg/dL; and during the entire study duration, with 38.5 mg/dL, without an additional hypoglycemia burden. This has occurred with a marked decrease in the first 6 months of treatment, a decrease that continued in the following 6 months, with a slower downward slope. The results of this study showed a more significant reduction in fasting blood glucose levels compared to the AWARD-3 trial, where the decrease was approximately 30 mg/dL [20,21].

Treatment with GLP-1 RAs was also associated with improvements in the lipid profile of the included patients, with a statistically significant difference between the initiation of the medication and 12 months later, as follows: a lower LDLc with an average of 8 mg/dL, a lower TG with an average of 19 mg/dL, a lower nonHDLc with an average of 3.8 mg/dL, and a higher HDLc with an average of 4 mg/dL, or, summed up, a cardioprotective improvement in atherogenic dyslipidemia, as also shown in the SELECT trial published in November 2023, although the causal effect cannot be assessed without a comparator arm [26]. Other favorable results concern other components of metabolic syndrome, namely blood pressure values, both systolic and diastolic, which decreased significantly in the 12 months of treatment with GLP-1 RAs, and also serum uric acid levels, which were significantly reduced, similarly to the results of the same SELECT randomized controlled trial and also to the results of several meta-analyses with this class of antihyperglycemic agents, although each was carried out with a completely different design not directly comparable with this real-world sample [26,27,28,29].

Lastly, although UACR did not change significantly in this study, the eGFR decrease was slower than the one expected for a similar population without a GLP-1 RA in their treatment regimen, results comparable to the ones of the FLOW trial, which highlighted a less steep decline in renal function in patients treated with injectable semaglutide [30]. However, considering that our study did not include a control group, it can only lead to descriptive associations, in contrast to the FLOW trial, where nephroprotection of injectable semaglutide can be affirmed.

### 4.2. Strengths and Weaknesses

This study analyzes the impact of treatment with GLP-1 RAs on glycemic values, HbA1c, weight, insulin resistance, and other cardio-reno-metabolic risk factors in patients with T2D and offers insights regarding a less-studied specific population, namely Romanian individuals (Central–Eastern European population) with this metabolic condition. Considering that T2D is a complex disease in which multiple genetic, environmental, and behavioral risk factors influence individuals’ susceptibility, this study focusing on a specific population offers valuable insights that can be further used in daily medical practice [31].

The weaknesses of this research include the retrospective design of the study and the absence of a control group, which both limit the possibility of identifying causal relationships between treatment with GLP-1 RA and suggested beneficial effects in this population, since residual confounding cannot be ruled out. Another limitation is caused by the scarce information regarding some of these potential confounding factors, including the other types of medication used by the subjects such as antihyperglycemic, antihypertensive, or lipid-lowering therapies, details that will be included in future studies. Regarding the statistical analysis of the data, small effect sizes can raise questions about clinical versus statistical significance, questions that could be resolved through future studies including a larger population observed for a longer duration of time. Additionally, information regarding lifestyle changes (such as dietary interventions or physical activity) and details regarding hospitalization, certain comorbidities, drug dosage, adherence, adverse events, tolerability, and treatment discontinuation rates were not consistently available; nevertheless, they will be included in future research.

### 4.3. Relevance of the Findings and Future Perspectives

This study highlights the importance of early initiation of treatment with GLP-1 RAs in patients with T2D in order to improve their cardio–reno–metabolic risk. Furthermore, it emphasizes the need for an early and multifactorial approach for these patients in order to prevent, to detect in a timely manner, and to treat possible chronic degenerative complications and comorbidities frequently encountered in such a setting, choosing, according to each case, an antihyperglycemic agent with proven beneficial pleiotropic effects. Future research that could assess in more detail the impact of GLP-1 RAs should include a greater number of subjects, with or without T2D but with excessive weight, while also including multiple insulin resistance, inflammation, and hepatic biomarkers, with an additional focus on body composition, quality of life, and mental health evaluations.

## 5. Conclusions

Treatment with GLP-1 RAs in Romanian patients with T2D is associated with a sustainably improved cardiovascular–kidney–metabolic risk profile, ameliorated glycemic control, reduced weight, lower insulin resistance, and slower kidney disease progression. These effects reinforce the recommendation of GLP-1 RA as a key therapeutic choice early in the personalized and multifactorial management of these patients in order improve their health-related outcomes.

## Figures and Tables

**Table 1:** Characteristics of the studied sample before initiating GLP-1 RA therapy.

Variable	Overall	Men	Women	*p*
Number (%)	150 (100%)	71 (47.3%)	79 (52.7%)	-
Age (years) ^a^	62.00 [56.00; 67.00]	62.00 (76.47)	62.00 (74.62)	0.749
T2D duration (years) ^a^	14.00 [6.00; 17.00]	14.00 (70.29)	15.00 (80.17)	0.163
Weight (kg) ^a^	99.50 [90.00; 112.00]	102.00 (84.43)	97.00 (67.46)	0.016 *
BMI (kg/m^2^) ^a^	35.00 [32.00; 39.00]	34.00 (70.04)	36.00 (80.39)	0.145
HbA1c (%) ^a^	8.70 [7.80; 9.93]	8.70 (76.91)	8.76 (74.22)	0.705
FPG (mg/dL) ^a^	189.00 [150.00; 245.00]	200.00 (78.55)	186.00 (72.75)	0.414
TC (mg/dL) ^a^	173.00 [150.60; 206.00]	167.60 (73.66)	173.80 (77.12)	0.623
LDLc (mg/dL) ^a^	95.00 [80.00; 121.00]	95.00 (76.38)	96.00 (74.70)	0.814
TG (mg/dL) ^a^	173.00 [139.00; 231.00]	174.00 (76.27)	173.00 (74.80)	0.836
HDLc (mg/dL) ^a^	38.00 [34.00; 43.00]	36.00 (60.01)	41.00 (89.41)	<0.0001 *
eGFR (mL/min/1.73 m^2^) ^a^	85.85 [69.00; 100.00]	93.00 (80.98)	83.00 (70.56)	0.142
UACR (mg/g) ^a^	22.00 [10.30; 46.00]	24.21 (81.52)	20.30 (70.08)	0.107
SBP (mmHg) ^a^	140.00 [130.00; 146.00]	140.00 (74.73)	140.00 (76.18)	0.836
DBP (mmHg) ^a^	80.00 [78.00; 85.00]	80.00 (74.78)	80.00 (76.14)	0.844
UA (mg/dL) ^b^	5.20 ± 1.24	5.52 ± 1.30	4.92 ± 1.11	0.002 *

^a^ Continuous variable with non-parametric distribution. Results are presented as median, [interquartile range], and (average rank). The *p*-value was calculated using the Mann–Whitney test. ^b^ Continuous variable with Gaussian distribution. Results are presented as mean ± standard deviation. The p-value was calculated using the unpaired Student’s *t*-test. * *p*-value < 0.05 (statistical significance). GLP-1 RA—glucagon-like peptide-1 receptor agonist, T2D—type 2 diabetes, BMI—body mass index, HbA1c—hemoglobin A1c, FPG—fasting plasma glucose, TC—total cholesterol, LDLc—low-density lipoprotein cholesterol, TG—triglyceride, HDLc—high-density lipoprotein cholesterol, eGFR—estimated glomerular filtration rate, UACR—urinary albumin/creatinine ratio, SBP—systolic blood pressure, DBP—diastolic blood pressure, UA—uric acid.

**Table 2:** Chronic degenerative complications of diabetes and comorbidities before initiating GLP-1 RA therapy.

Variable ^a^	Overall	Men	Women	*p*
Diabetic retinopathy	45 (30.0%)	21 (29.6%)	24 (30.4%)	0.915
Diabetic polyneuropathy	73 (48.7%)	37 (52.1%)	36 (45.6%)	0.425
Peripheral artery disease	35 (23.3%)	24 (33.8%)	11 (13.9%)	0.004 *
Chronic kidney disease	82 (54.7%)	35 (49.3%)	47 (59.5%)	0.211
Coronary artery disease	61 (40.7%)	32 (45.1%)	29 (36.7%)	0.299
Stroke	26 (17.3%)	15 (21.1%)	11 (13.9%)	0.246
Heart failure	42 (28.0%)	20 (28.2%)	22 (27.8%)	0.965
Hypertension	126 (84.0%)	61 (85.9%)	65 (82.3%)	0.545

**^a^** Results are presented as number (percentage of total). The *p*-value was calculated using the chi-squared test. * *p*-value < 0.05 (statistical significance). GLP-1 RA—glucagon-like peptide-1 receptor agonist.

**Table 3:** Evolution of weight and glycemic control during treatment with GLP-1 RA.

Parameter	Initially (a)	After 6 Months (b)	After 12 Months (c)	Difference a–b	Difference b–c	Difference a–c	Difference a–b–c
	Median	Median	Median	*p* ^y^	*p* ^y^	*p* ^y^	*p* ^z^
Weight ^x^	99.50 [90; 112]	95.00 [86; 108]	93.00 [86; 102]	<0.0001 *	<0.0001 *	<0.0001 *	<0.00001 *
	Paired difference			−4.00	−2.00	−6.00	
BMI ^x^	35.00	33.15	33.00	<0.0001 *	0.0005 *	<0.0001 *	<0.00001 *
	Paired difference			−1.10	−0.80	−2.00	
HbA1c ^x^	8.70 [7.80; 9.93]	7.95 [7.05; 9.0]	7.90 [7.0; 9.0]	<0.0001 *	0.0234 *	<0.0001 *	<0.00001 *
	Paired difference			−0.73	−0.00	−0.80	
FPG ^x^	189.00	164.00	146.00	<0.0001 *	<0.0001 *	<0.0001 *	<0.00001 *
	Paired difference			−14.50	−11.50	−38.50	

^x^ Continuous variable with non-parametric distribution. Results are presented as median ± [interquartile range]. ^y^ The *p*-value was calculated using the Wilcoxon test (paired samples). ^z^ The *p*-value was calculated using the Friedman test. * *p*-value < 0.05 (statistical significance). GLP-1 RA—glucagon-like peptide-1 receptor agonist, BMI—body mass index, HbA1c—hemoglobin A1c, FPG—fasting plasma glucose.

**Table 4:** Evolution of parameters after 12 months with GLP-1 RA.

Parameter	Initially	After 12 Months	Paired Difference	*p*
LDLc (mg/dL)	95.50	88.50	−8.00	<0.0001 *
HDLc (mg/dL)	38.00	42.00	4.00	<0.0001 *
TG (mg/dL)	173.50	154.00	−19.00	<0.0001 *
SBP (mmHg)	140.00	130.00	−5.00	<0.0001 *
DBP (mmHg)	80.00	80.00	0.00	0.042 *
UA (mg/dL)	5.00	4.85	−0.35	<0.0001 *
UACR (mg/g)	22.00	19.04	−1.92	0.481
eGFR (mL/min/1.73 m^2^)	85.85	85.50	−1.17	<0.0001 *

Continuous variable with non-parametric distribution. Results are presented as median. The *p*-value was calculated using the Wilcoxon test (paired samples). * *p*-value < 0.05 (statistical significance). GLP-1 RA—glucagon-like peptide-1 receptor agonist, LDLc—low-density lipoprotein cholesterol, HDLc—high-density lipoprotein cholesterol, TG—triglycerides, SBP—systolic blood pressure, DBP—diastolic blood pressure, UA—uric acid, UACR—urinary albumin–creatinine ratio, eGFR—estimated glomerular filtration rate.

**Table 5:** Evolution of insulin resistance biomarkers after 12 months of treatment with GLP-1 RA.

Parameter	Initially	After 12 Months	*p*
Tg/HDLc	4.35 [3.34; 6.24]	3.46 [2.58; 4.98]	<0.0001 *
TyG	4.24 [4.06; 4.36]	4.05 [3.91; 4.19]	<0.0001 *

Continuous variable with non-parametric distribution. Results are presented as median [interquartile range]. The *p*-value was calculated using the Wilcoxon test (paired samples). * *p*-value < 0.05 (statistical significance). GLP-1 RA—glucagon-like peptide-1 receptor agonist, Tg/HDLc—triglyceride/high-density lipoprotein cholesterol ratio, TyG—triglyceride–glucose index.

## Data Availability

The original contributions presented in this study are included in the article. Further inquiries can be directed to the corresponding author.

## Abbreviations

The following abbreviations are used in this manuscript:

ASCVD	Atherosclerotic cardiovascular disease
BMI	Body mass index
CKD	Chronic kidney disease
CKD-EPI	Chronic Kidney Disease Epidemiology Collaboration
CV	Cardiovascular
CVOT	Cardiovascular outcome trial
DBP	Diastolic blood pressure
eGFR	Estimated glomerular filtration rate
FPG	Fasting plasma glucose
GLP-1 RA	Glucagon-like peptide-1 receptor agonist
HbA1c	Hemoglobin A1c
HDLc	High-density lipoprotein cholesterol
LDLc	Low-density lipoprotein cholesterol
SBP	Systolic blood pressure
TC	Total cholesterol
TG	Triglycerides
TyG	Triglyceride–glucose index
T2D	Type 2 diabetes
UA	Uric acid
UACR	Urinary albumin–creatinine ratio
